# The effect of ArcA on the growth, motility, biofilm formation, and virulence of *Plesiomonas shigelloides*

**DOI:** 10.1186/s12866-021-02322-y

**Published:** 2021-10-04

**Authors:** Junxiang Yan, Yuehua Li, Xueqian Guo, Xiaochen Wang, Fenxia Liu, Ang Li, Boyang Cao

**Affiliations:** 1 TEDA Institute of Biological Sciences and Biotechnology, Nankai University, No.23, Hongda Street, Tianjin Economic and Technological Development Area, Tianjin, 300457 China; 2 Key Laboratory of Molecular Microbiology and Technology of the Ministry of Education, Nankai University, No.23, Hongda Street, Tianjin Economic and Technological Development Area, Tianjin, 300457 China; 3 Tianjin Key Laboratory of Microbial Functional Genomics, TEDA College, Nankai University, No.23, Hongda Street, Tianjin Economic and Technological Development Area, Tianjin, 300457 China; 4 State Key Laboratory of Medicinal Chemical Biology, College of Pharmacy and Tianjin Key Laboratory of Molecular Drug Research, Nankai University, Haihe Education Park, 38 Tongyan Road, Tianjin, 300353 China

**Keywords:** *Plesiomonas shigelloides*, ArcA, Growth, Motility, Biofilm formation, Virulence

## Abstract

**Background:**

The anoxic redox control binary system plays an important role in the response to oxygen as a signal in the environment. In particular, phosphorylated ArcA, as a global transcription factor, binds to the promoter regions of its target genes to regulate the expression of aerobic and anaerobic metabolism genes. However, the function of ArcA in *Plesiomonas shigelloides* is unknown.

**Results:**

In the present study, *P. shigelloides* was used as the research object. The differences in growth, motility, biofilm formation, and virulence between the WT strain and the Δ*arcA* isogenic deletion mutant strain were compared. The data showed that the absence of *arcA* not only caused growth retardation of *P. shigelloides* in the log phase, but also greatly reduced the glucose utilization in M9 medium before the stationary phase. The motility of the Δ*arcA* mutant strain was either greatly reduced when grown in swim agar, or basically lost when grown in swarm agar. The electrophoretic mobility shift assay results showed that ArcA bound to the promoter regions of the *flaK*, *rpoN*, and *cheV* genes, indicating that ArcA directly regulates the expression of these three motility-related genes in *P. shigelloides*. Meanwhile, the ability of the Δ*arcA* strain to infect Caco-2 cells was reduced by 40%; on the contrary, its biofilm formation was enhanced. Furthermore, the complementation of the WT *arcA* gene from pBAD33-*arcA*^+^ was constructed and all of the above features of the pBAD33-*arcA*^+^ complemented strain were restored to the WT level.

**Conclusions:**

We showed the effect of ArcA on the growth, motility, biofilm formation, and virulence of *Plesiomonas shigelloides*, and demonstrated that ArcA functions as a positive regulator controls the motility of *P. shigelloides* by directly regulating the expression of *flaK*, *rpoN* and *cheV* genes.

**Supplementary Information:**

The online version contains supplementary material available at 10.1186/s12866-021-02322-y.

## Background

*Plesiomonas shigelloides*, a gram-negative, rod-shaped bacterium that causes foodborne intestinal infections [1], can cause gastroenteritis, including acute secretory gastroenteritis, an invasive shigellosis-like disease, and a cholera-like illness [2–4]. Escobar et al. found that co-infections of *P. shigelloides* with either rotavirus or pathogenic *Escherichia coli* were 16.2-fold (95% confidence interval (CI) 5.5–62.3) and 13.8-fold (95% CI 3.3–69.3) more likely to result in diarrhea, respectively [5]. Extra intestinal infections, such as meningitis, bacteremia, and pseudoappendicitis, including skin and soft tissue infections, are also associated with *P. shigelloides* infection [6–8]. Fresh and estuarine water are considered the natural environments of *P. shigelloides*, which is often isolated from fish and other seafood [9].

*P. shigelloides* can grow under both aerobic and anaerobic conditions [10, 11]. The enzymes required for catabolism under aerobic and anaerobic conditions are substantially different; therefore, at the same time, to respond to the availability of oxygen, it is necessary to regulate the expression of genes related to cell functions, such as nutrient absorption and excretion systems, biosynthetic pathways, and macromolecule synthesis [12]. The Arc two-component signal transduction system, comprising the kinase sensor ArcB and its cognate response regulator ArcA, is one of the mechanisms that enable *E. coli* to adapt to changing oxygen availability [13, 14]. ArcB is activated in the form of a simplified electron carrier under conditions of hypoxia and energy provided by ATP. It has three cytoplasmic domains, and the autophosphorylation of His292 in the H1 domain, followed by transfer of the phosphate group to Asp576 in the D1 domain, then to His717 in the H2 domain [15], and finally to Asp54 in ArcA results in phosphorylation of ArcA [16], which activates ArcA to promote or repress the expression of Arc-regulated genes.

A previous study indicated that about 1139 genes in the *E. coli* K-12 genome are regulated either directly or indirectly by ArcA [17]. Under anaerobic conditions, ArcA inhibits the expression of genes required for aerobic metabolism, energy generation, amino acid transport, and fatty acid transport [18]. Another transcription factor involved in controlling anaerobic gene expression and facilitating bacterial adaptation to anaerobic conditions is FNR (fumarate and nitrate respiration) [19]. A comparison of the ArcA and FNR regulons showed that 303 genes were regulated by both proteins [17]. Jiang et al. found that citrate utilization in an anaerobic environment in *E. coli* is under direct control of FNR via the CitA-CitB system and under indirect control by ArcA [20]. A recent study showed that ArcA overexpression in aerobic conditions results in downregulation of respiratory pathways and enhanced growth rates on glycolytic substrates of *E. coli*, coinciding with acetate excretion and increased carbon uptake rates [21].

ArcA also controls chemotaxis and motility, contributing to the pathogenicity of *E. coli* [22]. Kato et al. determined that the Δ*arcA* mutant displayed a motility-defective phenotype and ArcA is necessary for the expression of *fliA* [23]. Furthermore, in *Salmonella enterica sv. Typhimurium*, the Δ*arcA* mutant was also non-motile and lacked flagella [24]. Biofilms are sessile bacterial communities that predominate in nature, and may form wherever a solid surface is in contact with a liquid [25]. Many opportunistic pathogens are capable of biofilm formation. *E. coli* dominates biofilms found on urethral catheters, and has also been isolated from percutaneous trans-hepatic catheters [26, 27]. Previous studies on certain enterobacteria and non-enterobacteria have also reported the relationship between ArcA and biofilms. For example, Hengge proposed that ArcA has a regulatory role between the sigma factor RpoS and biofilm formation [28]. Xi et al. found that the response regulator ArcA enhances biofilm formation in a vpsT-dependent manner under anaerobic conditions in *Vibrio cholerae* [29]. In addition, studies on *Actinobacillus pleuropneumoniae* and *Haemophilus parasuis* also suggested that ArcA regulates the formation of biofilms positively [30, 31]. However, in *Porphyromonas gingivali*, Wu et al. showed that ArcA inhibits FimA production and inhibits biofilm formation [32].

In addition to ArcA being related to cell metabolism, biosynthesis, and motility, many studies have provided evidence that ArcA is related to virulence. For example, a recent study found that ArcA of *E. coli* K12, which causes human meningitis, downregulates the expression of sRNA-17 to benefit bacterial survival in blood and the penetration of the blood-brain barrier [33]. Moreover, ArcA is also required for the toxicity of *Salmonella typhimurium*, *Vibrio cholerae*, *Haemophilus influenzae*, and *Actinobacillus pleuropneumoniae* [34–38].

The effects of ArcA in *P. shigelloides* are unknown; therefore, the present study aimed to determine the correlation between ArcA and growth, motility, biofilm formation, and virulence in *P. shigelloides*.

## Results

### Phylogenetic analysis of ArcA

**Fig. 1:**
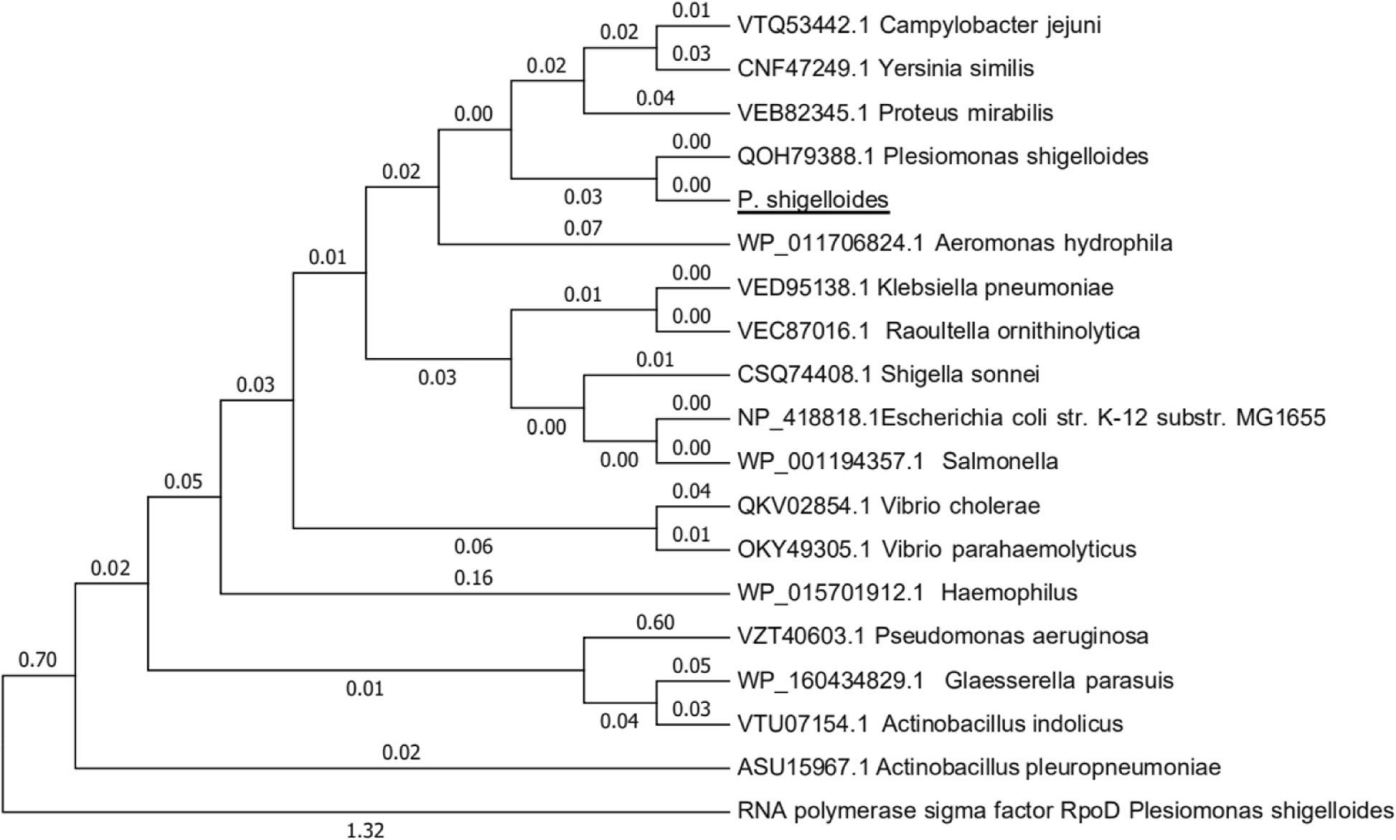
An unrooted phylogenetic tree constructed using the neighbor joining method based on ArcA amino acid sequences. Bootstrap values were based on 1000 replications and only values greater than 50% are shown

The two-component system response regulator ArcA of *P. shigelloides* is comprised of 238 amino acids. A phylogenetic tree based on ArcA amino acid sequences was constructed using the neighbor-joining method and plotted by MEGA 6.0. Bootstrap analysis was carried out based on 1000 replicates. The RopD protein of *P. shigelloides* was selected as the outgroup control. A dendrogram consisting of 17 species of bacteria, including some common human gut bacteria, was constructed. The comparison results showed that ArcA is conserved in all the selected bacteria. ArcA of *P. shigelloides* is relatively close to those from *Proteus* and *Aeromonas*, but far from those from *Actinobacillus* and *Pseudomonas* (Fig. 1).

### Identification of the deletion and complementation of *arcA*

**Fig. 2:**
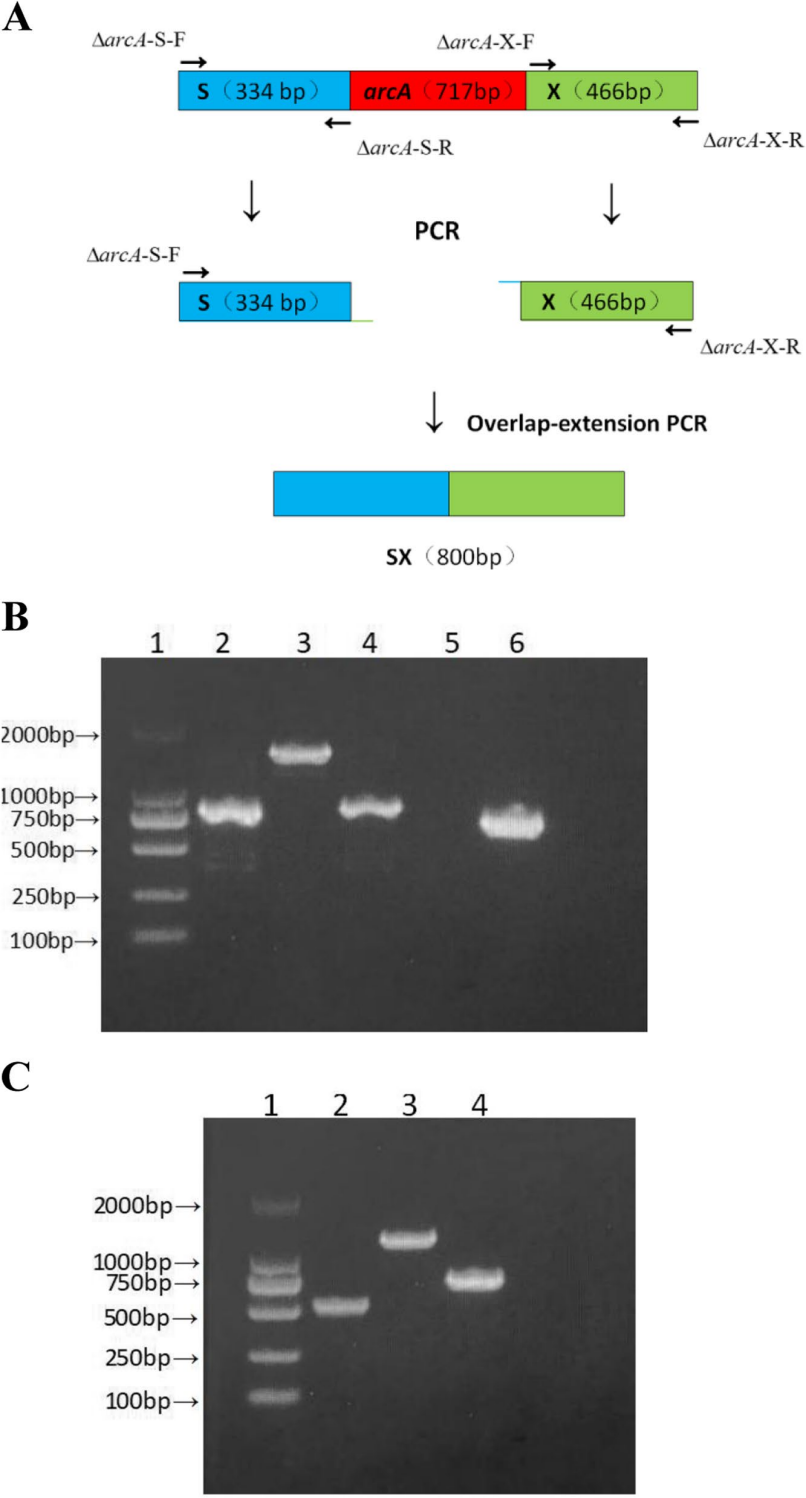
Confirmation of the deletion and complementation of *arcA* in *P. shigelloides*. **A** Graphical process of the deletion of the *arcA* gene. **B** PCR detection of the product. 1, DL2000 DNA marker; 2, PCR fragment of SX; 3, PCR amplicon of S-*arcA*-X from the WT genomic DNA; 4, PCR amplificon of SX from the Δ*arcA* genome DNA; 5, PCR amplification of *arcA* from the Δ*arcA* genome DNA; 6, PCR amplification of *arcA* from the WT genome DNA. **C** 1, DL2000 DNA marker; 2, PCR amplification of pBAD33-UD from the pBAD33 plasmid; 3, PCR amplification of pBAD33-U-*arcA*-D from the *arcA*^*+*^ complementation strain; 4, PCR amplification of *arcA* from the genomic DNA of the complementation strain

A schematic illustration of the overlap-extension PCR method used for deletion of *arcA* is shown in Fig. 2A. The deletion and identification of *arcA* is showed in Fig. 2B, in which SX (800 bp) and S-*arcA-*X (1517 bp) are the controls for ArcA^−^ and ArcA^+^, respectively. The Δ*arcA* isogenic deletion mutant strain was obtained (Lane 4 in Fig. 2B). To further confirm the result, we designed *arcA* identification primers, *arcA*-F and *arcA*-R, to amplify the *arcA* gene from the genomes of Δ*arcA* and the WT, respectively. The PCR reaction generated a negative signal with Δ*arcA* and a positive one with the WT (717 bp).

The complementation of *arcA* is shown in Fig. 2C. The pBAD33-UD (529 bp) is a negative control. After complementation, pBAD33-U-*arcA*-D (1246 bp) and *arcA* (717 bp) were both amplified with the correct sizes.

### ArcA affected the microaerobic growth of *P. shigelloides*

**Fig. 3:**
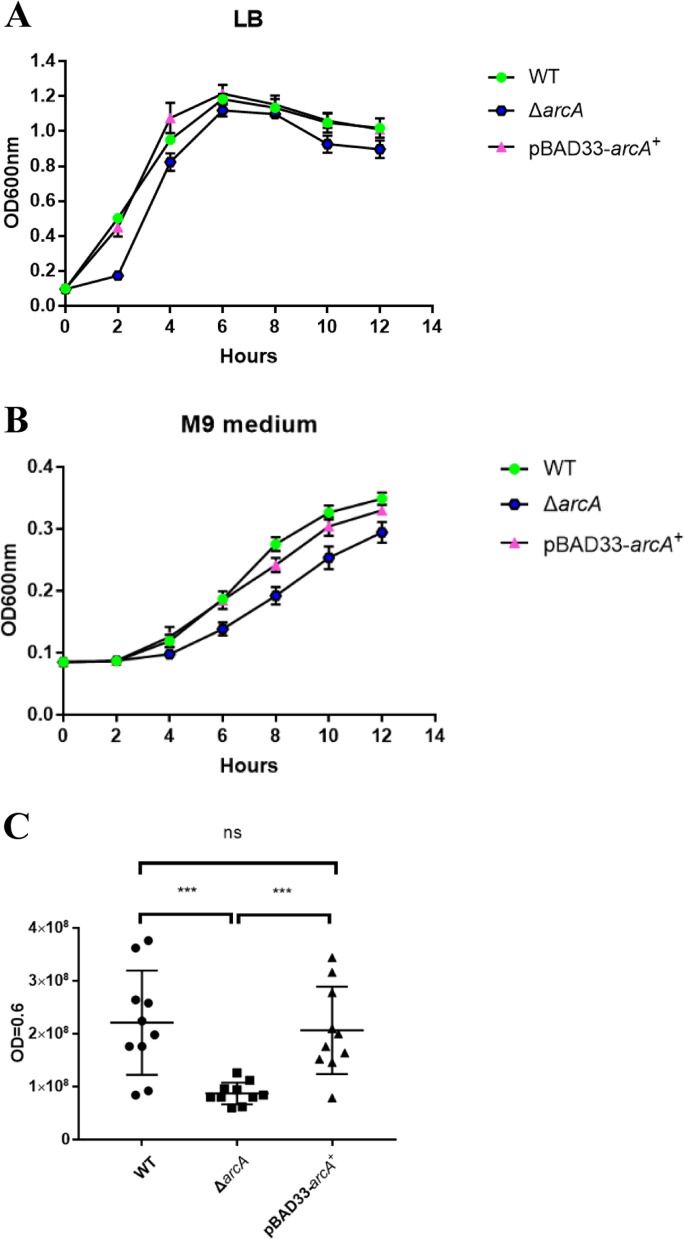
Deletion of *arcA* affected the growth of *P. shigelloides* in either LB medium or M9 medium under microaerobic conditions. **A** Bacterial strains were grown in LB and **B** M9 medium containing only glucose as a carbon source under microaerobic conditions, and the optical density at 600 nm (OD_600_) was monitored. **C** Bacterial strains were grown to OD_600_ = 0.6, and the plate colony counting method was used to count the three strains separately. The experiments were performed three times in quadruplicate. Significant differences were indicated by asterisks (***P < 0.001)

In this study, we used LB liquid medium and M9 minimal medium with only glucose as a carbon source to verify the role of ArcA in the growth and reproduction of *P. shigelloides*. When grown in LB liquid, the growth of Δ*arcA* slightly lagged behind that of the WT in the lag and log phases before 6 h, and the growth was completely restored to the WT level upon complementation with *arcA* (Fig. 3A). When grown in the M9 minimal medium with only glucose as the carbon source, the growth difference between Δ*arcA* and WT were obvious, and the Δ*arcA* mutants lagged behind the growth of WT before WT entered the stable phase at 12 h. Growth in M9 plus glucose was completely restored to the WT level upon complementation with *arcA* (Fig. 3B), which indicated that ArcA affects the uptake and utilization of glucose by *P. shigelloides*. In addition, the colony forming units were counted for the WT, Δ*arcA* and pBAD33-*arcA*^+^ strains at OD_600_ = 0.6, which showed that there was a 2.6-fold reduction for Δ*arcA* compared with that for the WT (Fig. 3C).

### ArcA controls the motility of *P. shigelloides* by directly regulating the expression of *flaK*, *rpoN* and *cheV* genes

**Fig. 4:**
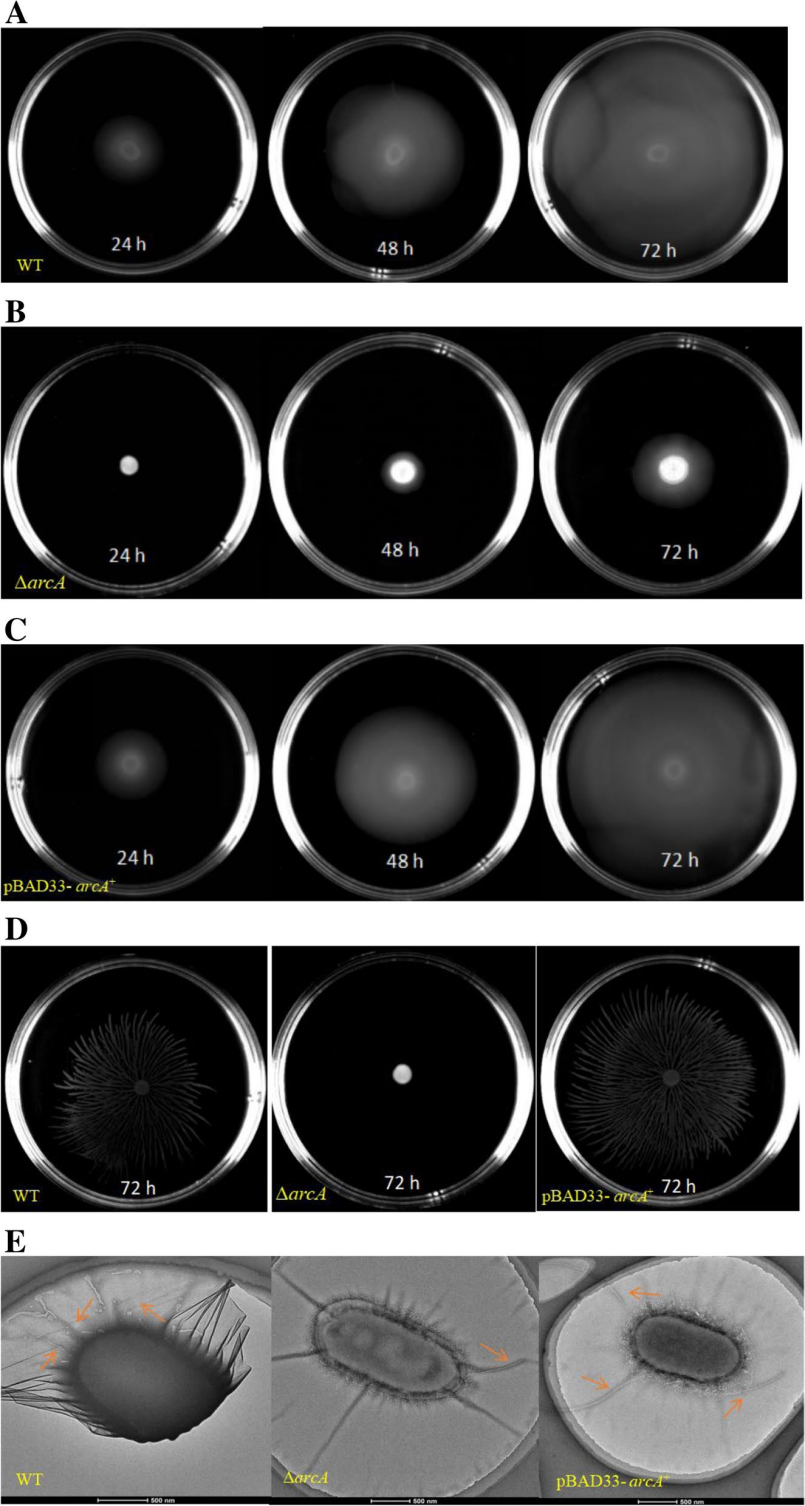
Motility of the WT, Δ*arcA* and pBAD33-*arcA*^+^ strains. **A** The WT, **B** Δ*arcA*, and **C** pBAD33-*arcA*^+^ strains grown in swimming agar plates for 24 h, 48 h and 72 h, respectively. **D** From left to right, the WT, Δ*arcA* and pBAD33-*arcA*^+^ strains grown in swarming agar plates for 72 h. **E** TEM visualization of the flagella produce by the WT, Δ*arcA* and pBAD33-*arcA*^+^ strains from left to right. The hollow bacterial flagella were pointed by the colored arrows

In addition to ArcA being related to the growth and metabolism of *P. shigelloides*, we also found that ArcA is related to motility. The WT, Δ*arcA* and pBAD33-*arcA*^+^ strains were freshly cultured, transferred to both swimming and swarming agar plates, and incubated at 25 °C for 24–72 h. When grown in swimming agar plates, the motility of the Δ*arcA* strain was markedly reduced compared with the WT. There was almost no obvious movement traces after the Δ*arcA* strain was grown for 24 h, and it spread by 2.8 cm when cultured for 72 h (Fig. 4B). In contrast, the WT and pBAD33-*arcA*^+^ strains had overgrown the plates under the same conditions at 72 h (Fig. 4A and C). The movement data of the strains in swimming agar plates are listed in Table 1. Moreover, when grown in swarming agar plates, the motility of the Δ*arcA* strain was totally lost, and there was no significant change even it was cultured for 72 h. Interestingly, the WT and pBAD33-*arcA*^+^ strains showed irregular trajectories similar to radials when grown in swarming agar plates (Fig. 4D), which was rarely mentioned in previous studies. The flagella produced by the WT, Δ*arcA*, and pBAD33-*arcA*^+^ strains were observed by TEM. Compared to the Δ*arcA* mutant strain with a single flagellum, the WT and pBAD33-*arcA*^+^ strains showed the typical three-four flagella (Fig. 4E). TEM results indicated that the lack of ArcA attenuates the flagella synthesis in *Plesiomonas shigelloides*.

**Table 1:** The movement diameter of the strains in swimming agar plates

Strains	Swimming		
	24 h	48 h	72 h
WT	2.3 cm	4.8 cm	7.4 cm
∆*arcA*	0.6 cm	1.5 cm	2.8 cm
pBAD33-*arcA*^+^	2.6 cm	5.2 cm	7.6 cm

**Fig. 5:**
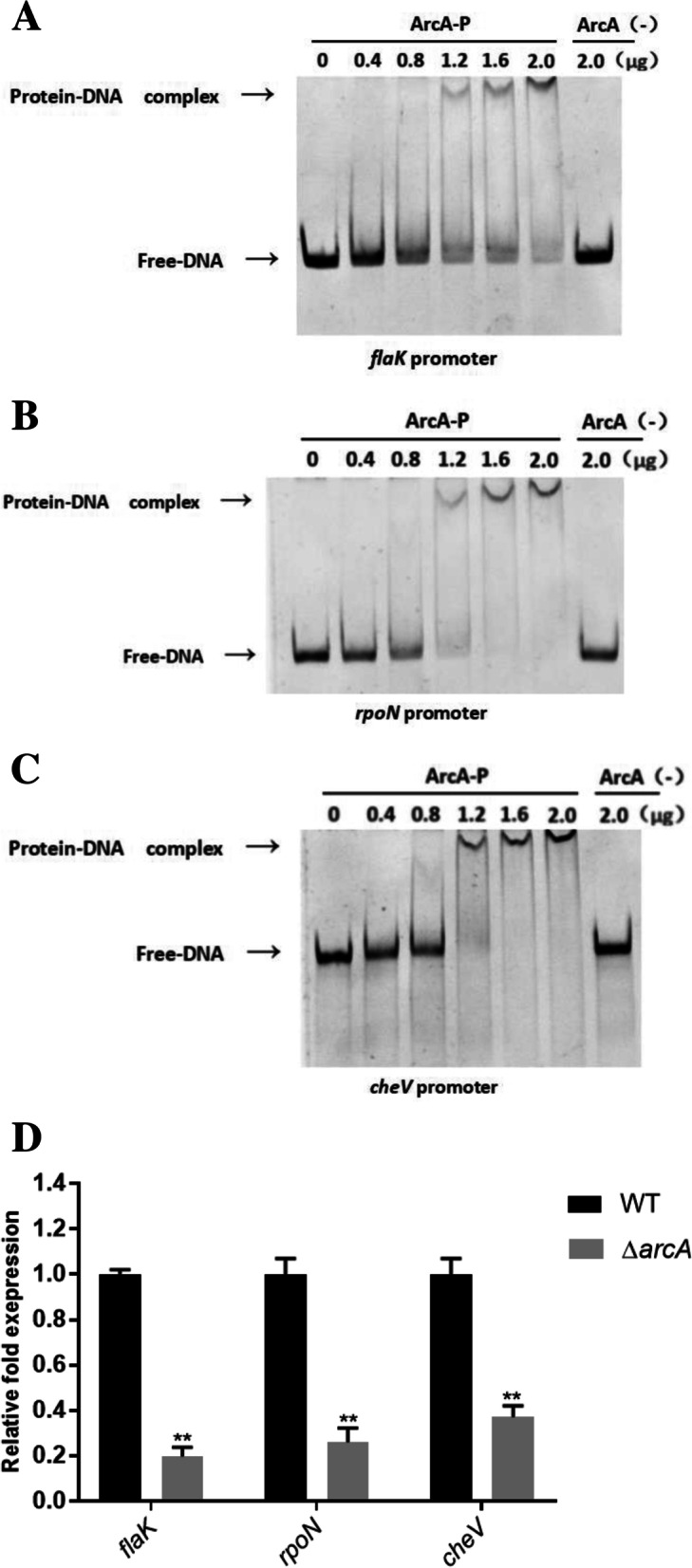
ArcA controls the motility of *P. shigelloides* by directly binding the promoter regions of *flaK*, *rpoN* and *cheV* genes. **A** The EMSA of phosphorylated ArcA protein and the *flaK* promoter. **B** The EMSA of phosphorylated ArcA protein and the *rpoN* promoter. **C** The EMSA of phosphorylated ArcA protein and the *cheV* promoter. The concentration of phosphorylated ArcA protein was increased gradually with the non-phosphorylated ArcA as a negative control (non-ArcA-P). **D** The mRNA level of *flaK*, *rpoN* and *cheV* of the WT and Δ*arcA* mutant. Significant differences were indicated by asterisks (**P < 0.01)

A previous search for the putative ArcA binding sites at the flagella gene cluster promoter region was performed using Virtual Footprint 3.0. The analysis predicted the presence of ArcA binding sites in the promoter regions of *flaK*, *rpoN* and *cheV* genes (see Fig. S1A to C). To confirm a direct interaction between ArcA and the predicted binding sites, ArcA-His_6_ fusion protein was expressed and purified (Fig. S1D), three genes promoter region were generated by PCR and used to perform EMSA with phosphorylated ArcA (ArcA-P) and non-phosphorylated ArcA (non-ArcA-P) as the negative control. The complex of protein and DNA with ArcA-P were observed when incubated with *flaK*, *rpoN* and *cheV* promoter fragments (Fig. 5A, B and C). The negative control (non-ArcA-P) generated no shifts even at high protein concentration (2.0 μg). Then we performed the qRT-PCR and found that the expression of *flaK*, *rpoN* and *cheV* decreased approximately 5.6-, 4.3-, and 2.7-fold in the Δ*arcA* mutant compared to the WT (Fig. 5D). The data indicated that ArcA functions as a positive regulator controls the motility of *P. shigelloides* by directly regulating the expression of *flaK*, *rpoN* and *cheV* genes.

### ArcA negatively regulates *P. shigelloides* biofilm formation

**Fig. 6:**
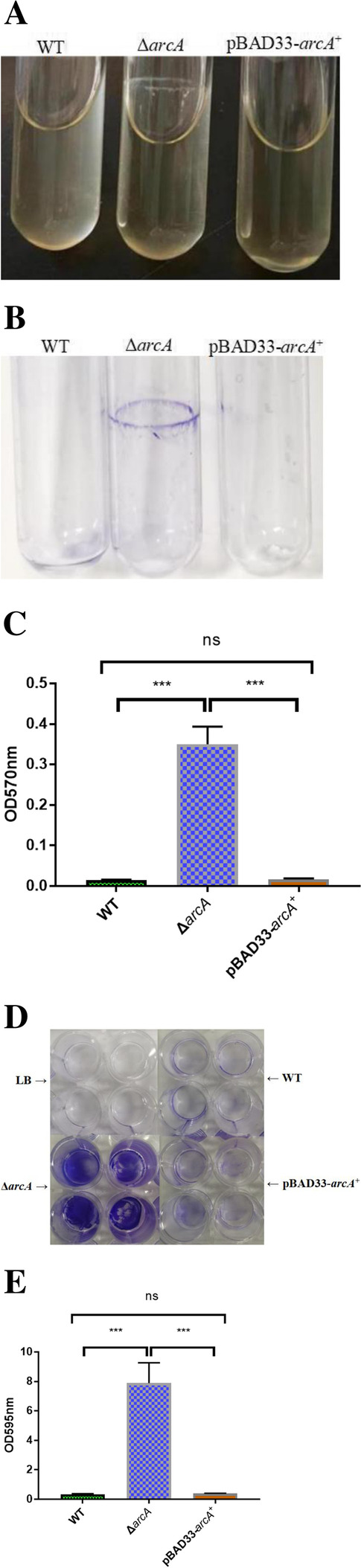
Biofilm formation assays with both glass tubes and 24-well culture plates. **A** The biofilm formation of the WT, Δ*arcA* and pBAD33-*arcA*^+^ strains cultured in the glass tubes. **B** The biofilm mass determined by staining surface-attached cells with crystal violet. **C** The biofilm formation was measured at OD_570_. **D** The biofilm formation of the WT, Δ*arcA* and pBAD33-*arcA*^+^ strains cultured in 24-well plates. **E** The biofilm formation was measured at OD_595_. Significant differences were indicated by asterisks (***P < 0.001)

The biofilm formation assays were performed by both glass-tubes and 24-well plates. When the WT, Δ*arcA* and pBAD33-*arcA*^+^ strains were cultured in a glass-tube, the results showed that the WT could not form a biofilm. By contrast, the Δ*arcA* strains could form a biofilm circle at the surface of liquid, which was visible to the naked eyes. After *arcA* was complemented in the deletion strains, the biofilm formation ability disappeared (Fig. 6A). Furthermore, purple crystal violet staining was observed for the residue in the tubes containing the Δ*arcA* strain but in not the glass tubes that had contained the other two strains (Fig. 6B). In addition, we also quantitatively measured the biofilm formation ability and the results indicated that biofilm formation of Δ*arcA* (OD_570_ approximately 0.35) was 21.56-fold higher than that in the WT (Fig. 6C). In addition, for the bacteria were cultured in the 24-well culture plates, with LB only as the negative control. Compared to the Δ*arcA* strain, which formed an obvious biofilm at the bottom of the wells, only a small amount of residues was observed for the WT and pBAD33-*arcA*^+^ strains after being stained (Fig. 6D). The quantitative measurement results showed that biofilm formation ability of the Δ*arcA* (OD_595_ approximately 7.86) was 23.01-fold higher than that in the WT (Fig. 6E). The data of the above two biofilm formation assays indicated that ArcA fundamentally inhibits biofilm formation in *P. shigelloides*.

### ArcA enhances the invasion of Caco-2 cells in *P. shigelloides*

**Fig. 7:**
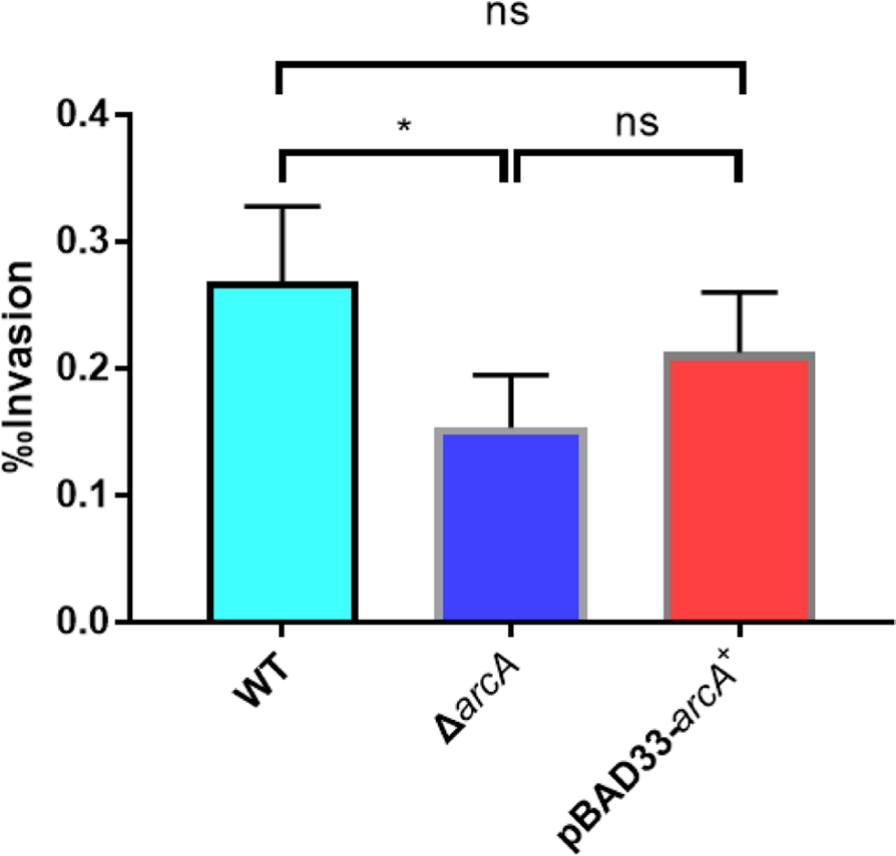
The invasion capability of the WT, ∆*arcA*, and pBAD33-*arcA*^+^ strains into Caco-2 cells. Results were performed using analysis of variance (ANOVA) of four independent assays. Significant differences were indicated by asterisks (*P < 0.05)

Compared with the *P. shigelloides* WT, the Δ*arcA* mutant showed a 40% reduction in its capacity to invade Caco-2 cells. In contrast to the biofilm results, the pBAD33-*arcA*^+^ complementation strain could restore the invasive ability only partially, failing to reach the same level as the WT (Fig. 7). The assay was repeated four times and the difference in invasion capabilities between the WT and ∆*arcA* was statistically significant (*p* = 0.0186). The data demonstrated that ArcA could enhance the ability to invade eukaryotic cells in *P. shigelloides*.

## Discussion

As a facultative anaerobe, *P. shigelloides* can obtain energy under anaerobic or aerobic conditions through phosphorylation reactions related to electron transfer. The ArcAB binary regulatory system and the global regulatory protein FNR (ferric nitrate reductase) have been proven to play a major regulatory role in the metabolic process in response to changes in oxygen [39, 40]. Most of the known ArcA target genes of *E. coli* are related to aerobic respiration metabolism, and the DNA binding activity of ArcA is regulated by the reversible phosphorylation of ArcB [41]. Park et al. identified a total of 229 differentially expressed operons under anaerobic growth conditions by ChIP, among which ArcA has a direct regulatory effect on 85 of them by bioinformatic analysis [42]. At present, the role of ArcA in bacterial energy metabolism is not very clear. However, based on our comparison of the growth of *P. shigelloides* and the Δ*arcA* strain in the two media (LB and M9), it can be seen that ArcA has an impact on the metabolism of nutrients. When the Δ*arcA* strain was grown in M9 minimal medium with glucose as the carbon source, the glucose utilization rate was significantly lower than that of the WT before reaching the stable period. These results indicated that there is a certain connection between ArcA and the nutrition and energy metabolism of *P. shigelloides*.

In addition to the regulation of oxidative metabolism in bacteria, our data also confirmed that ArcA is related to bacterial motility. *P. shigelloides* is the unique member of the Enterobacteriaceae family that is able to produce polar flagella when grown in liquid medium and lateral flagella when grown in solid or semisolid media [43]. Previous studies have shown that *P. shigelloides* contained two different gene clusters, one exclusively for the lateral flagella biosynthesis and the other one containing the biosynthetic polar flagella genes [44]. The *P. shigelloides* polar flagella gene regions occupy higher similarity to those reported in *Vibrio Parahemolyticus* and *Aeromonas hydrophila* than the regions in *E. coli* or *S. typhimurium* [44, 45]. The primary regulatory factor of the polar flagella region of *P. shigelloides* is FlaK, not the FlhDC in *E. coli*. *P. shigelloides* lateral gene cluster is almost identical to the one of *A. hydrophila* [46]. However, no LafK ortholog could be detected in *P. shigelloides* even though the *lafK* gene has been reported in all the lateral gene clusters in the Enterobacteriaceae [46, 47]. In addition, we found that the trajectory of *P. shigelloides* in swarming agar plates was radial rather than circular, which was also different from the swarming motion shape of *P. dendritiformis* type-C [48] and *Pseudomonas aeruginosa* [49]. We suggest that the higher agar concentration of the swarming agar plates induced the production of lateral flagella in *P. shigelloides*, and resulted in a radial movement trajectory. Taken together, polar and lateral flagella transcriptional hierarchy in the *P. shigelloides* could represents a different Gammaproteobacteria model. Here, we provide evidence that ArcA could control the motility of *P. shigelloides* by directly regulating the expression of *flaK*, *rpoN* and *cheV* genes, and next we will focus on the flagella regulation mechanism of *P. shigelloides* in the future study.

Bacterial biofilms are bacteria that adhere to the surface of non-biological or active tissues in order to adapt to the living environment, and are coated in the mucus heterogeneous polymer matrix produced by themselves, forming a bacterial group that grows in a different way from planktonic bacteria [50]. Bacterial adhesion is the first step of bacterial biofilm formation. Previous studies reported that the *groEL* operon is related to adhesion and cell toxicity in *P. shigelloides* [51]. Edward et al. compared the genome sequence of 11 strains of *Plesiomonas shigelloides* and found that some strains contained biofilm forming proteins PgaA, PgaB and PgaC. However, subsequent experiments proved that *Plesiomonas shigelloides* strain EE2 can be formed even without these proteins. This indicated that *P. shigelloides* uses other mechanisms to regulate the formation of biofilms [52]. We found *pgaC* in the genome sequence of the *P. shigelloides* strain used in this experiment, but did not find *pgaA* and *pgaB*. At the same time, the WT showed almost no biofilm formation ability. However, after the *arcA* gene was deleted, the biofilm formation ability of the Δ*arcA* mutant strain was significantly enhanced, which indicated that ArcA has a relatively strong ability to inhibit the formation of *P. shigelloides* biofilms under normal conditions. Therefore, it is necessary to explore the relationship between ArcA and biofilm formation in subsequent studies. In the present study, our data also showed that ArcA is related to the virulence of *P. shigelloides*. Compared with the WT, the Δ*arcA* mutant showed a 40% reduction in infectivity of Caco-2 cells. However, the specific regulation mechanism is remains unclear. In addition, flagella [53–55], adhesin [56], Type 1 fimbriae [57], and curled fimbriae [58–61] are also essential for bacterial biofilm formation and virulence. They mediate the adhesion, movement, and chemotaxis of bacteria to help them seek advantages and avoid harm.

## Conclusions

In this work, we report the roles of ArcA in *P. shigelloides*, and the data showed that ArcA could control the motility of *P. shigelloides* by directly regulating the expression of *flaK*, *rpoN* and *cheV* genes. And, the phenotype experiments in this study is significant for further discovering the specific links between ArcA and *P. shigelloides* in terms of growth, metabolism, biofilm formation, and virulence. Our results also laid a foundation to reveal the pathogenic mechanisms of *P. shigelloides*.

## Materials and methods

### Bacterial strains, growth conditions, and plasmids

The bacterial strains, as well as the plasmids used, are listed in Table 2. Bacteria were grown in tryptic soy broth (TSB), tryptic soy agar (TSA); and Luria-Bertani (LB) liquid, solid, and semi-solid medium at 37 °C statically or in a shaking incubator, or at 25 °C statically. If necessary, media were supplemented with ampicillin (25 μg/ml), chloramphenicol (25 μg/ml) or kanamycin (50 μg/ml).

**Table 2:** Bacterial strains and plasmids used in this study

Strains/plasmids	Genotype or relevant characteristics^a^	Source or reference
*Plesiomonas shigelloides* strains		
G5884	Wild type, serotype O45:H2^b^	CNCTC^b^ Aer 44/89
△*arcA*	*arcA* gene deletion mutant of G5884	This study
Δ*arcA*/pBAD33-*arcA*^+^	Δ*arcA* containing pBAD33 carrying *arcA* ORF with its own promoter	This study
*E. coli* strains		
DH5α λ*pir*	Transformation host	Lab collection
S17–1 λ*pir*	Tp^R^ Sm^R^*recA*, *thi*, *pro*, *hsdR*-M + RP4: 2-Tc:Mu: Km Tn7 λ*pir*, Kmr, Smr, Tpr	[62]
BL21(DE3)	Host strain for protein expression	Lab collection
BL21/ pET28a-*arcA*^+^	BL21(DE3) with pET28a carrying the *arcA* ORF; Km^r^	This study
Plasmids		
pRE112	Widely used gene knocked vector, with onT RP4, Cm^r^	[63]
pBAD33	Arabinose inducible expression vector, CmR	[64]
pET28a	T7 expression vector; Km^r^	Lab collection
pRE112-*arcA*^*−*^	pRE112 containing the homologous arms of *arcA* gene of G5884, Cmr	This study
pBAD33-*arcA*^+^	pBAD33 with complete *arcA*	This study
pET28a-*arcA*^+^	pET28a carrying the *arcA* gene; Km^r^	This study

^a^*r* Resistant

^b^*CNCTC* Czech National Collection of Type Cultures, the Czech Republic

### Deletion and complementation studies of *arcA*

In this study, an effective and precise conjugate transfer process mediated by the suicide vector pRE112 was used to make deletion mutations in the *arcA* gene of *P. shigelloides* [65]. The complementation strains was constructed by introducing the recombinant vector pBAD33-*arcA*^+^ into the Δ*arcA* strain via electroporation. DNA sequencing were used to confirm the presence of the correct deletion mutations and complementation strains. And all primers used in this study are shown in Table 3.

**Table 3:** Primers used in this study

Name	Sequence (5′–3′)	Amplifified fragment
Primers for construction of mutants		
∆*arcA*-S-F	GCTCTAGATTTTGGCTAAATCTGTGTGC	∆*arcA*-S (334 bp)
∆*arcA*-S-R	GGTCAATTGCGTGGGCCAACTGCTTGCGCCTTC	
∆*arcA*-X-F	GAAGGCGCAAGCAGTTGGCCCACGCAATTGACC	∆*arcA*-X (466 bp)
∆*arcA*-X-R	GGGGTACCTTTGAGAAGGTGATGCCG	
		∆*arcA*-SX (800 bp)
*arcA*-F	ATGCAAACCCCGCACATT	*arcA* (717 bp)
*arcA*-R	TTACTCTTCCAGCTCGCCG	
Primers for identification of plasmid		
pRE112-U-F	CACTGTTCGTCCATTTCCG	pRE112-UD (567 bp)
pRE112-D-R	TTCGTCTCAGCCAATCCCT	
		pRE112-U-*arcA*-D (1284 bp)
pBAD33-U-F	AACAAAGCGGGACCAAAG	pBAD33-UD (529 bp)
pBAD33-D-R	AGAGCGTTCACCGACAAA	
		pBAD33-U-*arcA*-D (1246 bp)
pET28a-U-F	TAATACGACTCACTATAGGG	pET28a-UD (318 bp)
pET28a-D-R	GCTAGTTATTGCTCAGCGG	
		pET28a-U-*arcA*-D (1035 bp)
Primers for construction of complemented strain		
pBAD33-*arcA*^+^-F	GGGGTACCATGCAAACCCCGCACATT	*arcA*^+^ (733 bp)
pBAD33-*arcA*^+^-R	GCTCTAGATTACTCTTCCAGCTCGCCG	
Primers for protein cloning		
pET28a-*arcA*^+^-F	CGGGATCCATGCAAACCCCGCACATT	*arcA*^+^ (733 bp)
pET28a-*arcA*^+^-R	CCCTCGAGTTACTCTTCCAGCTCGCCG	
Primers for qRT-PCR		
16S rRNA-F	GGCAGCAGTGGGGAATATTG	275 bp
16S rRNA-R	AGTTGAGCTCGGGGATTTCA	
qRT-flaK-F	CTGGTGGAGCGGTTGGTTAT	254 bp
qRT-flaK-R	GGACCTTCATGACCAGCACA	
qRT-rpoN-F	AAACGGTGGAAATGCACGAA	158 bp
qRT-rpoN-R	GACTAATGCTCGAATGGCCG	
qRT-cheV-F	AGCGCACACAATTAGTCGGA	210 bp
qRT-cheV-R	CAATCGCCAAGCTCATGTCG	
Primers for EMSA		
EMSA-flaK-F	ACGGAAAGTCTTGACACTGTG	309 bp
EMSA-flaK-R	TTTATGGCAGCGACTATAGC	
EMSA-rpoN-F	GTCGCCGAGTAGAAATTGCC	350 bp
EMSA-rpoN-R	CGTACTTCCCGGCTAAGC	
EMSA-cheV-F	GGTAATAGTTTGCCGTCCCG	378 bp
EMSA-cheV-R	TAACGTGCTACTCCCAGGG	

Underlined letters show Kpn1, Xba1, BamhI or XhoI restriction site

*S/X-F/R* The upstream and downstream primers for the upstream and downstream gene fragments of *arcA* in the O45 genome, *arcA*^*(+)*^*-F/R* Upstream and downstream primers of *arcA* gene, *U/D-F/R* Upstream and downstream sequencing primers of plasmid

### RNA isolation and quantitative real time PCR (qRT-PCR)

Total RNA was extracted using TRIzol® Reagent (Invitrogen, Waltham, MA, USA #15596-018) according to the manufacturer’s protocol. qRT-PCR analysis was conducted on an Applied Biosystems ABI 7500 sequence detection system with SYBR green fluorescence dye. The *P. shigelloides* 16S rRNA gene was used as the internal control for qRT-PCR, and relative expression levels were calculated as fold change values using the 2^-△△CT^ method. Each experiment was carried out in triplicate.

### Electrophoretic mobility shift assay (EMSA)

*E. coli* BL21 (DE3) with pET28a-*arcA*^+^ was grown in 200 ml of LB medium for 5 h at 30 °C, and protein expression was induced by adding 0.1 mM isopropyl beta-D-1-thio-galactopyranoside (IPTG). The ArcA-His_6_ fusion protein was purified using an Ni-NTA-Sefinose Column (Sangon Biotech, Shanghai, China #C600791) in accordance with the protocol provided by the manufacturer. Phosphorylation reactions of ArcA were carried out as described previously [20]. EMSAs were performed by adding increasing amounts of purified and phosphorylated ArcA-His_6_ fusion protein (0, 0.4, 0.8, 1.2,1.6 and 2.0 μg) to the DNA probe (50 ng) in binding buffer (100 mM Tris-HCl pH 7.5, 10 mM MgCl_2_, 2 mM DTT, 100 mM KCl, 10% glycerol) for 30 min at 37 °C. DNA–protein complexes were separated by 6% PAGE in 0.5 × TBE buffer (44.5 mM Tris, 44.5 mM boric acid, 1 mM EDTA, pH 8.0) at 160 V for 1 h. Gels were stained with GelRed for 10 min and imaged using a gel imaging system (GE Healthcare, Chicago, IL, USA).

### Dynamic growth of the WT, Δ*arcA* and pBAD33-*arcA*^+^ strains

The WT, Δ*arcA* and pBAD33-*arcA*^+^ bacterial strains were cultured overnight at 37 °C with shaking into sterile LB medium and until they reached an OD_600_ = 0.6. Then, the bacterial solution was added to five wells of a 96-well cell plate containing 200 μl of LB at a ratio of 1:200 per well. Fresh LB was added to the surrounding wells as a control. Finally, the prepared 96-well cell plate was placed in a Molecular Devices Spectra MAX 190 full-wavelength microplate reader (Molecular Devices, San Jose, CA, USA) to carry out the Dynamic growth experiment. The dynamic growth experiment for the WT, Δ*arcA* and pBAD33-*arcA*^+^ strains was also carried out in M9 medium, which contains only glucose as a carbon source. The temperature was controlled at 37 °C throughout the whole process. We conducted the experiments at three time points with five repetitions for each time.

### Motility assays

The motility assays were performed as described previously [66]. Freshly grown bacterial colonies were transferred using a sterile toothpick into the center of swarming agar or swimming agar plates. The swimming agar plates were incubated for 24–72 h at 25 °C and motility was examined by the migration of bacteria through the agar from the center toward the plate periphery. Additionally, according to experimental requirements, the swarming agar plates were incubated up for 72 h at 25 °C. We conducted the experiments at three time points with six repetitions for each time.

### Transmission electron microscopy (TEM)

TEM and negative staining used to visualize the flagella of the WT, Δ*arcA*, and pBAD33-*arcA*^+^ strains was as previously described [24].

### Biofilm assay

In this study, we carried out the biofilm formation assay as described previously [67, 68] with some modifications. The WT, Δ*arcA*, and pBAD33-*arcA*^+^ strains were grown overnight in TSB. The next day, the overnight bacterial solution was transferred to fresh TSB and the bacteria were grown to OD_600_ = 0.6. The bacteria were then subcultured in fresh LB liquid medium at 1:100 and inoculated into 10 × 75 mm borosilicate glass test tubes containing 3 ml of sterile LB, and incubated at 37 °C for 20 h without shaking. Subsequently, the tubes were rinsed with phosphate-buffered saline (PBS) and filled with 0.1% crystal violet stain. After 5 min, the tubes were rinsed and then photographed. The biofilm-associated crystal violet was resuspended in dimethyl sulfoxide (DMSO), and the OD_570_ of the resulting suspension was measured. In addition, we also applied a 24-well tissue culture plate for the biofilm formation assay [52] on the WT, Δ*arcA* and pBAD33-*arcA*^+^ strains. All experiments were performed at three time-points independently and each individual samples were assayed in four repetitions.

### Invasion assays

The invasion assay was carried out as described previously [69], with some modifications. Briefly, approximately 5 × 10^7^ bacterial cells were layered onto confluent monolayers of approximately 1 × 10^5^ Caco-2 cells (suspended in Dulbecco’s modified Eagle’s medium (DMEM)) per well in 24-well plates. The plates were centrifuged at 1000×*g* for 30 s to promote the sinking of bacteria, followed by incubation at 37 °C in 5% CO_2_ for 1 h. The monolayer washed extensively with PBS, and fresh, pre-warmed DMEM containing gentamycin (100 μg/ml) was added to kill extracellular bacteria. After 1 h of incubation, the monolayer was washed with PBS twice, and the cells were lysed with 0.1% Triton X-100 for 10 min. The released intracellular bacteria were enumerated using the plate counting method. The invasive ability was expressed as the percentage of the inoculum that survived the gentamycin treatment. We conducted the assay at four time points with six repetitions for each time.

### Statistical analysis

Statistical analysis of the data was performed using analysis of variance (ANOVA). A probability value (*P*) ≤ 0.05 was considered statistically significant (*** *p* ≤ .001; ** *p* ≤ .01; * *p* ≤ .05; ns indicates not significant). The construction of the ArcA evolutionary tree used the Molecular Evolutionary Genetics Analysis (MEGA 6.0) software package [70].

## Supplementary Information

**Additional file 1: Fig. S1.** A. Putative ArcA binding sites at the *flaK* promoter region. B. Putative ArcA binding sites at the *rpoN* promoter region. C. Putative ArcA binding sites at the *cheV* promoter region. D. The purity of the purified ArcA-His_6_ fusion protein was analyzed by 10% sodium dodecyl sulphate (SDS)-polyacrylamide gel electrophoresis.

**Additional file 2: Figure S2.** Confirmation of the deletion of *arcA* in *P. shigelloides.* 1, DL2000 DNA marker (The bands shown in the electrophoretic gel are as follows: 2000 bp, 1000 bp, 750 bp, 500 bp, 250 bp and 100 bp); 2, PCR fragment of SX (800 bp); 3, PCR amplicon of S-*arcA*-X (1284 bp) from the WT genomic DNA; 4, PCR amplificon of SX from the Δ*arcA* genome DNA; 5, PCR amplification of *arcA* from the Δ*arcA* genome DNA; 6, PCR amplification of *arcA* (717 bp) from the WT genome DNA. Notice: SX,the upstream and downstream homologous fragments of *arcA*; S-*arcA*-X, PCR amplicon of the upstream and downstream of *arcA* and *arcA*. Moreover, Fig. 2B in manuscript was cropped from Figure S2. **Figure S3.** Confirmation of the complementation of *arcA* in *P. shigelloides.* 1, DL2000 DNA marker; 2, PCR amplification of pBAD33-UD (529 bp) from the pBAD33 plasmid; 3, PCR amplification of pBAD33-U-*arcA*-D (1246 bp) from the *arcA*^*+*^ complementation strain; 4, PCR amplification of *arcA* from the genomic DNA of the complementation strain. Notice: pBAD33- UD, The fragment obtained by PCR amplification of pBAD33 plasmid using identification primers; pBAD33-U-*arcA*-D. The fragment obtained by PCR amplification of pBAD33-*arcA*^+^ strain using identification primers. Figure 2C in manuscript was cropped from Figure S3. **Figure S4.** The EMSA between phosphorylated ArcA protein and the *flaK* promoter.The concentration of phosphorylated ArcA protein (ArcA-P) increased gradually (0 to 2.0 μg), the non-phosphorylated ArcA was used as a negative control (ArcA (−)) and the amount of promoter DNA used in each reaction was 50 ng. Figure 5A in manuscript was cropped from Figure S4. **Figure S5.** The EMSA between phosphorylated ArcA protein and the *rpoN* promoter. The concentration of phosphorylated ArcA protein (ArcA-P) increased gradually (0 to 2.0 μg), the non-phosphorylated ArcA was used as a negative control (ArcA (−)) and the amount of promoter DNA used in each reaction was 50 ng. Figure 5B in manuscript was cropped from Figure S5. **Figure S6.** The EMSA between phosphorylated ArcA protein and the *cheV* promoter.The concentration of phosphorylated ArcA protein (ArcA-P) increased gradually (0 to 2.0 μg), the non-phosphorylated ArcA was used as a negative control (ArcA (−)) and the amount of promoter DNA used in each reaction was 50 ng. Figure 5C in manuscript was cropped from Figure S6.

## Data Availability

All data generated or analyzed during this study are included in this published article.

## Abbreviations

ArcA: Anoxic redox control cognate response regulator
WT: Wild-type
Δ*arcA*: *arcA* isogenic deletion mutant strain
pBAD33-*arcA*^+^: Complementation strain of *arcA*
LB: Luria-Bertani
M9: M9 medium which contains only glucose as a carbon source
PBS: Phosphate-buffered saline
EMSA: Electrophoretic mobility shift
