# Supplementary material for: The effect of ArcA on the growth, motility, biofilm formation, and virulence of Plesiomonas shigelloides
Source: BMC Microbiol. 2021 Oct 4;21:266. doi: 10.1186/s12866-021-02322-y (PMC8489083; doi:10.1186/s12866-021-02322-y)
Supplement: Supplementary file 1 — Additional file 1: Fig. S1. A. Putative ArcA binding sites at the flaK promoter region. B. Putative ArcA binding sites at the rpoN promoter region. C. Putative ArcA binding sites at the cheV promoter region. D. The purity of the purified ArcA-His6 fusion protein was analyzed by 10% sodium dodecyl sulphate (SDS)-polyacrylamide gel electrophoresis. [file 12866_2021_2322_MOESM1_ESM.docx]

**Fig. S1**

A Putative ArcA binding sites at the*flaK*promoter region


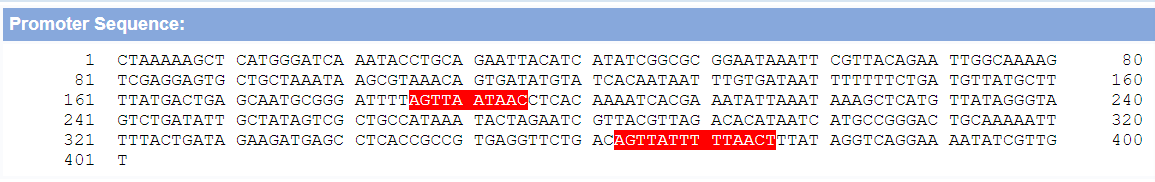


B Putative ArcA binding sites at the*rpoN*promoter region


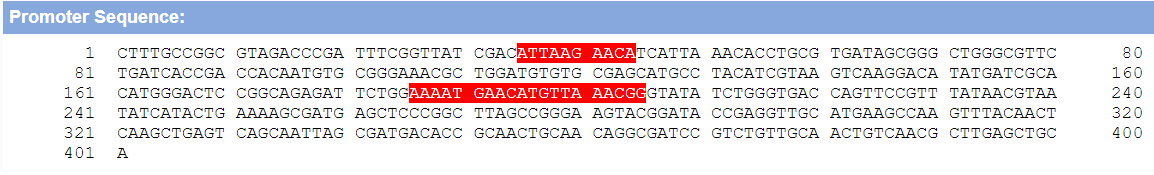


C Putative ArcA binding sites at the*cheV*promoter region


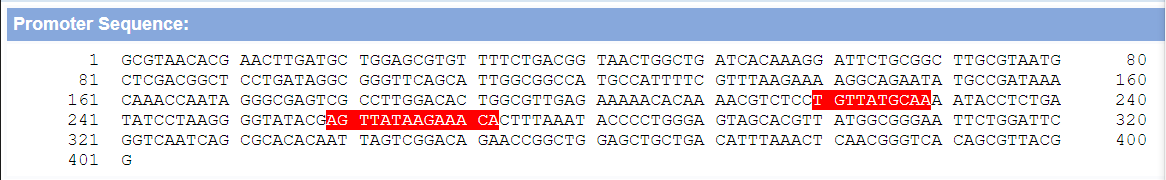


D The purity of the purified ArcA-His_6_ fusion protein was analyzed by 10% sodium dodecyl sulphate (SDS)-polyacrylamide gel electrophoresis.


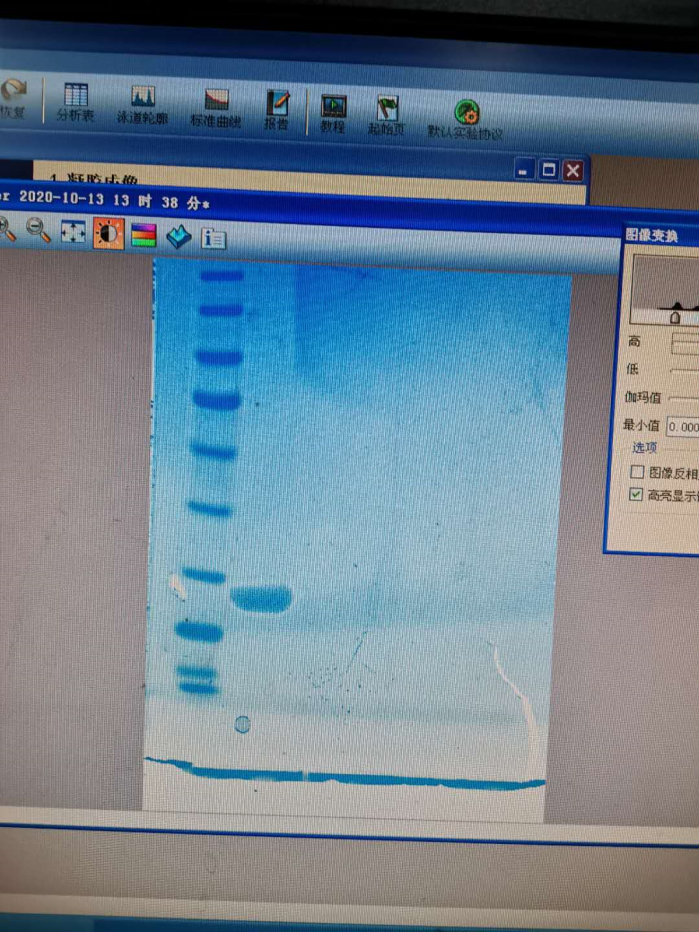


25 KD

35 KD
