# Supplementary material for: The effect of ArcA on the growth, motility, biofilm formation, and virulence of Plesiomonas shigelloides
Source: BMC Microbiol. 2021 Oct 4;21:266. doi: 10.1186/s12866-021-02322-y (PMC8489083; doi:10.1186/s12866-021-02322-y)
Supplement: Supplementary file 2 — Additional file 2: Figure S2. Confirmation of the deletion of arcA in P. shigelloides. 1, DL2000 DNA marker (The bands shown in the electrophoretic gel are as follows: 2000 bp, 1000 bp, 750 bp, 500 bp, 250 bp and 100 bp); 2, PCR fragment of SX (800 bp); 3, PCR amplicon of S-arcA-X (1284 bp) from the WT genomic DNA; 4, PCR amplificon of SX from the ΔarcA genome DNA; 5, PCR amplification of arcA from the ΔarcA genome DNA; 6, PCR amplification of arcA (717 bp) from the WT genome DNA. Notice: SX,the upstream and downstream homologous fragments of arcA; S-arcA-X, PCR amplicon of the upstream and downstream of arcA and arcA. Moreover, Fig. 2B in manuscript was cropped from Figure S2. Figure S3. Confirmation of the complementation of arcA in P. shigelloides. 1, DL2000 DNA marker; 2, PCR amplification of pBAD33-UD (529 bp) from the pBAD33 plasmid; 3, PCR amplification of pBAD33-U-arcA-D (1246 bp) from the arcA+ complementation strain; 4, PCR amplification of arcA from the genomic DNA of the complementation strain. Notice: pBAD33- UD, The fragment obtained by PCR amplification of pBAD33 plasmid using identification primers; pBAD33-U-arcA-D. The fragment obtained by PCR amplification of pBAD33-arcA+ strain using identification primers. Figure 2C in manuscript was cropped from Figure S3. Figure S4. The EMSA between phosphorylated ArcA protein and the flaK promoter.The concentration of phosphorylated ArcA protein (ArcA-P) increased gradually (0 to 2.0 μg), the non-phosphorylated ArcA was used as a negative control (ArcA (−)) and the amount of promoter DNA used in each reaction was 50 ng. Figure 5A in manuscript was cropped from Figure S4. Figure S5. The EMSA between phosphorylated ArcA protein and the rpoN promoter. The concentration of phosphorylated ArcA protein (ArcA-P) increased gradually (0 to 2.0 μg), the non-phosphorylated ArcA was used as a negative control (ArcA (−)) and the amount of promoter DNA used in each reaction was 50 ng. Figure 5B in manuscript was cropped fro [file 12866_2021_2322_MOESM2_ESM.docx]

**Figure. S2**

1

6

5

4

2

3


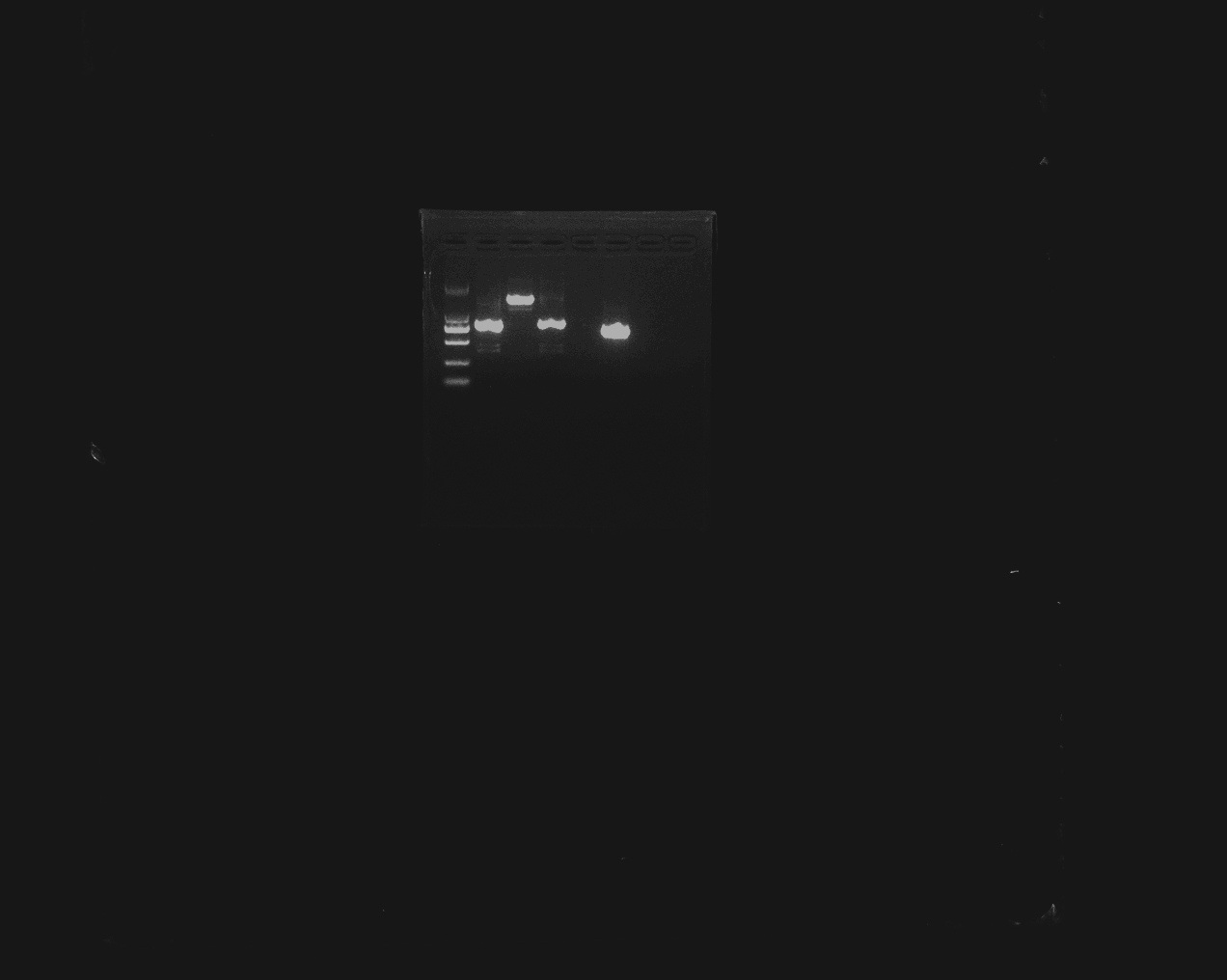


Figure. S2. Confirmation of the deletion of *arcA* in *P. shigelloides.* 1, DL2000 DNA marker（The bands shown in the electrophoretic gel are as follows: 2000 bp, 1000 bp, 750 bp, 500 bp, 250 bp and 100 bp）; 2, PCR fragment of SX (800 bp); 3, PCR amplicon of S‑*arcA*-X (1284 bp) from the WT genomic DNA; 4, PCR amplificon of SX from the Δ*arcA* genome DNA; 5, PCR amplification of *arcA* from the Δ*arcA* genome DNA; 6, PCR amplification of *arcA* (717 bp) from the WT genome DNA. **Notice:** SX,the upstream and downstream homologous fragments of *arcA*；S‑*arcA*-X,PCR amplicon of the upstream and downstream of *arcA* and *arcA* .Moreover, Figure. 2B in manuscript was cropped from Figure. S2.

**Figure. S3**

2

4

3

1


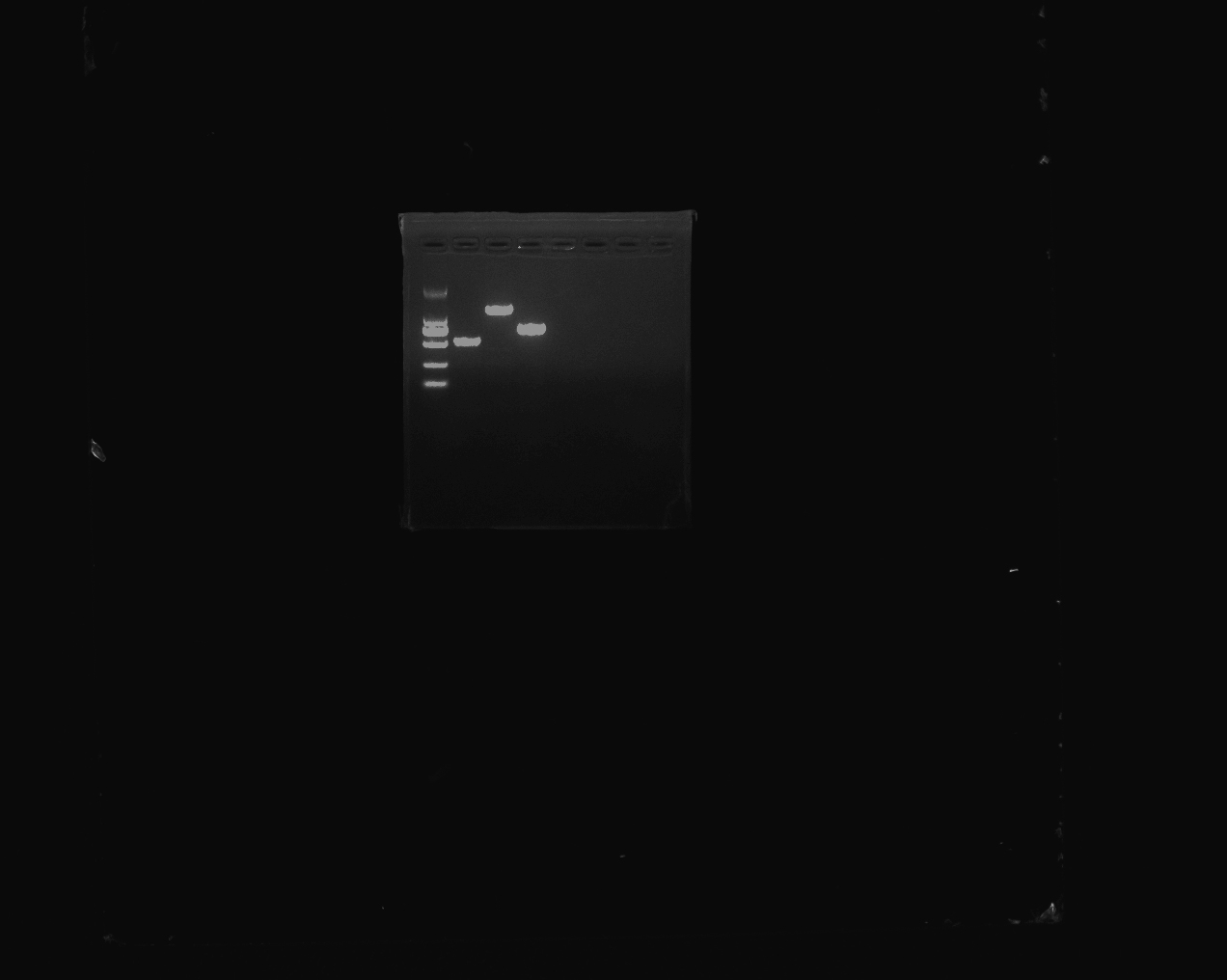


Figure. S3. Confirmation of the complementation of *arcA* in *P. shigelloides.* 1, DL2000 DNA marker; 2, PCR amplification of pBAD33-UD (529 bp) from the pBAD33 plasmid; 3, PCR amplification of pBAD33-U-*arcA*-D (1246 bp) from the *arcA^+^* complementation strain; 4, PCR amplification of *arcA* from the genomic DNA of the complementation strain. **Notice:** pBAD33- UD, The fragment obtained by PCR amplification of pBAD33 plasmid using identification primers；pBAD33-U-*arcA*-D,The fragment obtained by PCR amplification of pBAD33-*arcA*^+^ strain using identification primers.Figure. 2C in manuscript was cropped from Figure. S3.

**Figure. S4**


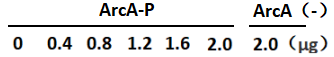

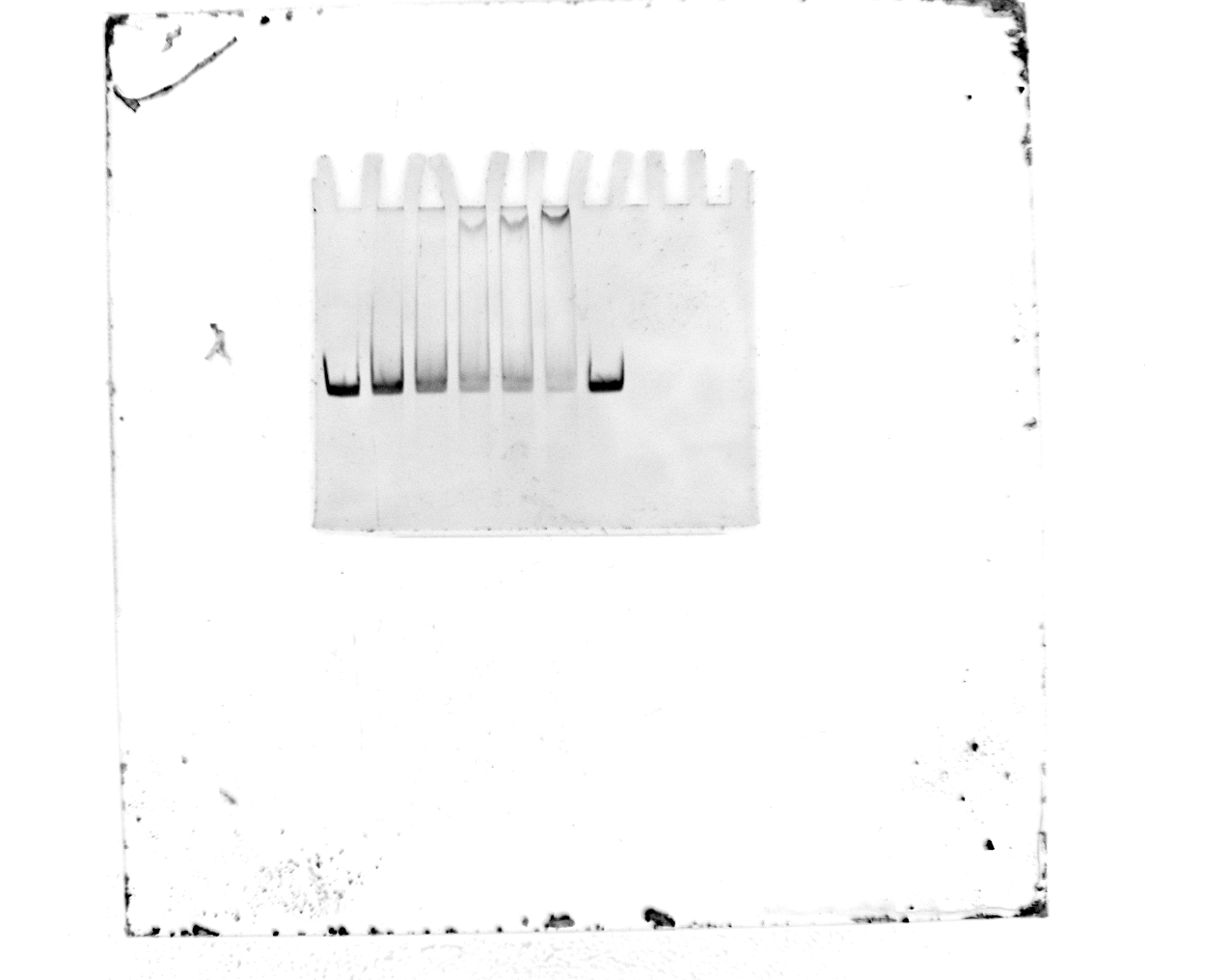

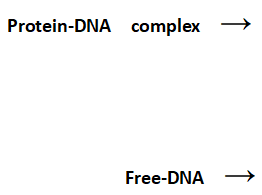

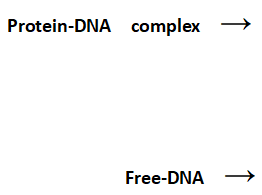


Figure. S4. The EMSA between phosphorylated ArcA protein and the *flaK* promoter.The concentration of phosphorylated ArcA protein (ArcA-P) increased gradually (0 to 2.0 μg) ，the non-phosphorylated ArcA was used as a negative control (ArcA (-)) and the amount of promoter DNA used in each reaction was 50 ng.Figure. 5A in manuscript was cropped from Figure. S4.

**Figure. S5**


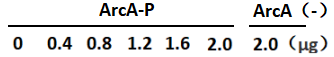

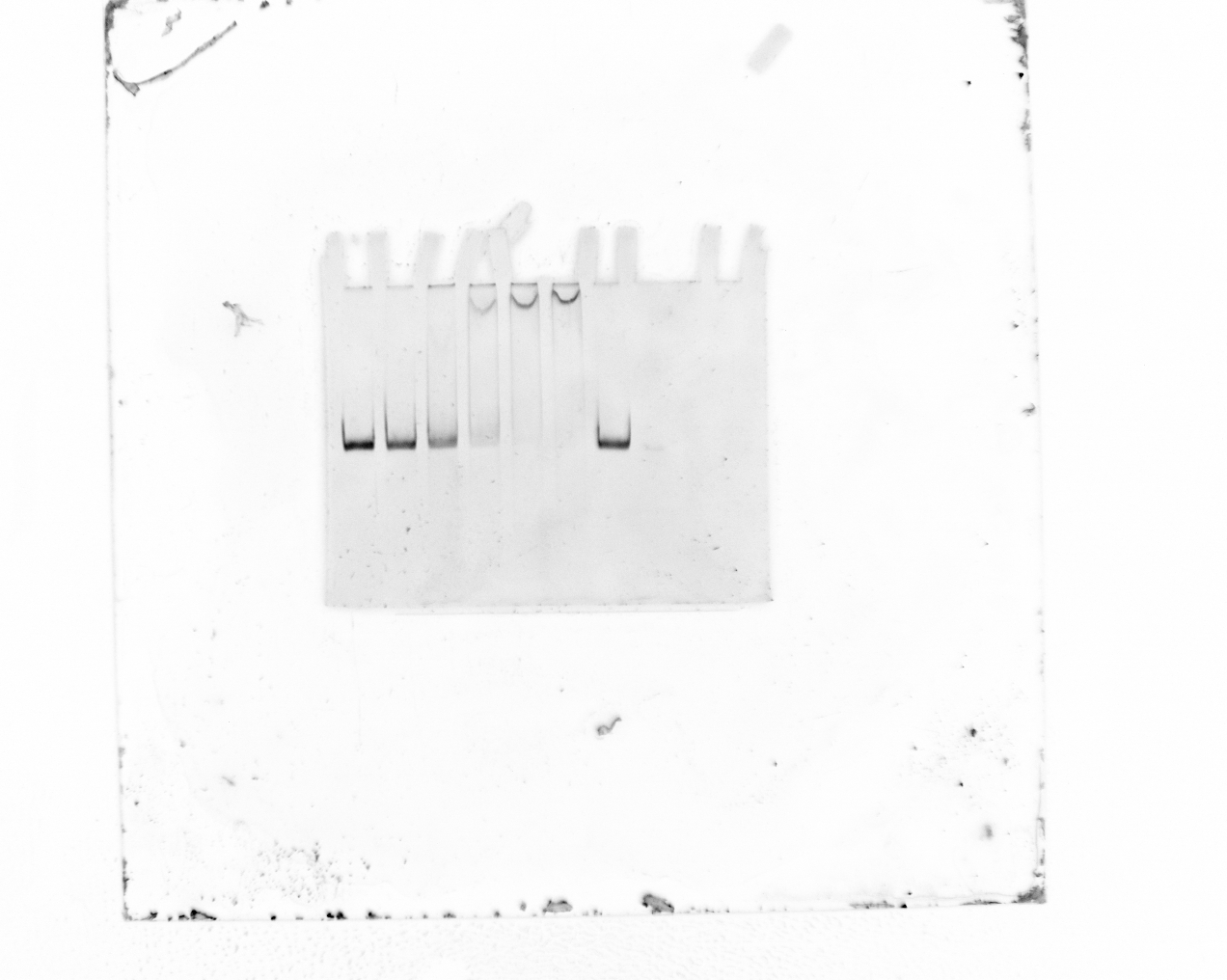

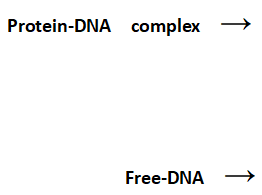

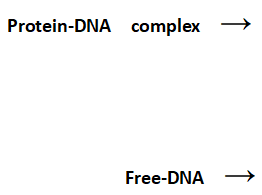


Figure. S5. The EMSA between phosphorylated ArcA protein and the *rpoN* promoter.The concentration of phosphorylated ArcA protein (ArcA-P) increased gradually (0 to 2.0 μg) ，the non-phosphorylated ArcA was used as a negative control (ArcA (-)) and the amount of promoter DNA used in each reaction was 50 ng.Figure. 5B in manuscript was cropped from Figure. S5.

**Figure. S6**


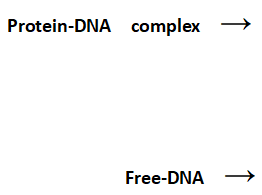

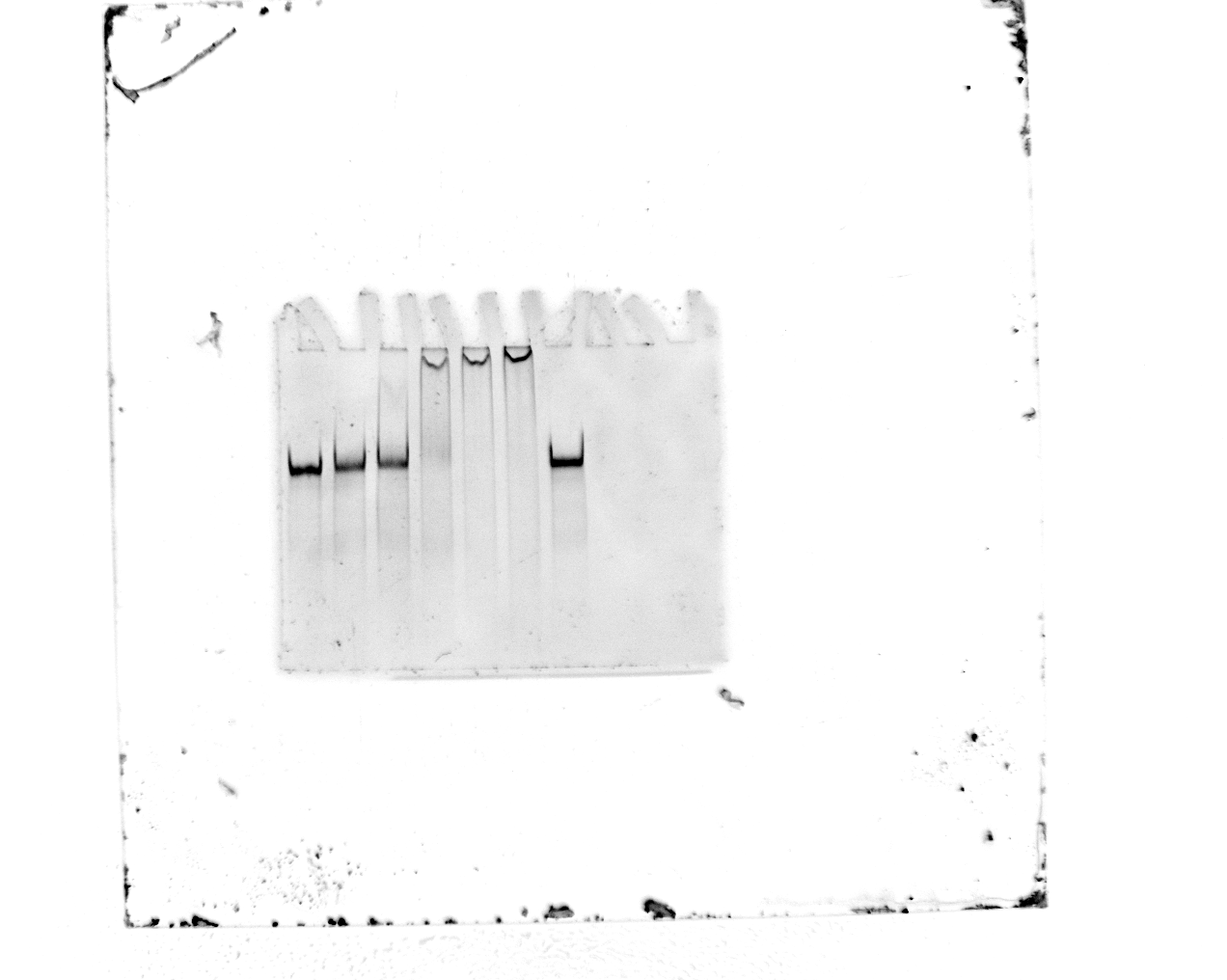

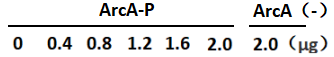

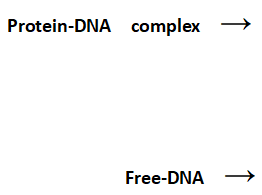


Figure. S6. The EMSA between phosphorylated ArcA protein and the *cheV* promoter.The concentration of phosphorylated ArcA protein (ArcA-P) increased gradually (0 to 2.0 μg) ，the non-phosphorylated ArcA was used as a negative control (ArcA (-)) and the amount of promoter DNA used in each reaction was 50 ng.Figure. 5C in manuscript was cropped from Figure. S6.
